# AP2/ERF transcription factors regulate the biosynthesis of terpenoids, phenolics, and alkaloids in plants

**DOI:** 10.1093/hr/uhaf280

**Published:** 2025-10-20

**Authors:** Qin Chen, Na Li, Xiuming Cui, Feng Ge

**Affiliations:** Yunnan Key Laboratory of Sustainable Utilization of Panax notoginseng Resources, Faculty of Life Science and Technology, Kunming University of Science and Technology, Kunming 650500, China; State Key Laboratory for Quality Ensurance and Sustainable Use of Dao-di Herbs, Beijing 100700, China; Yunnan Key Laboratory of Sustainable Utilization of Panax notoginseng Resources, Faculty of Life Science and Technology, Kunming University of Science and Technology, Kunming 650500, China; State Key Laboratory for Quality Ensurance and Sustainable Use of Dao-di Herbs, Beijing 100700, China; Yunnan Key Laboratory of Sustainable Utilization of Panax notoginseng Resources, Faculty of Life Science and Technology, Kunming University of Science and Technology, Kunming 650500, China; State Key Laboratory for Quality Ensurance and Sustainable Use of Dao-di Herbs, Beijing 100700, China; Yunnan Key Laboratory of Sustainable Utilization of Panax notoginseng Resources, Faculty of Life Science and Technology, Kunming University of Science and Technology, Kunming 650500, China; State Key Laboratory for Quality Ensurance and Sustainable Use of Dao-di Herbs, Beijing 100700, China

## Abstract

AP2/ERF transcription factors (TFs) constitute a large, plant-specific family that acts as a central hub integrating developmental and environmental signals to modulate the biosynthesis of secondary metabolites. These compounds, including terpenoids, phenolic compounds, and alkaloids, are vital for plant survival and are of immense value to human health and industry. This review provides a comprehensive synthesis of the molecular mechanisms by which AP2/ERF TFs regulate these crucial metabolic pathways. We systematically classify and dissect their regulatory modes, including direct binding to cis-elements (e.g. GCC-box, CE1, and DRE/CRT), indirect control via upstream signaling cascades, co-regulation through physical interactions with other TF families (e.g. MYB, bHLH, WRKY), and feedback regulation. We present numerous case studies across diverse plant species, highlighting both conserved principles and species-specific adaptations in the control of high-value natural products like artemisinin, tanshinones, anthocyanins, and nicotine. Furthermore, we discuss the emerging roles of AP2/ERF TFs in metabolic engineering and synthetic biology, and outline future research directions, emphasizing the application of multi-omics and CRISPR/Cas9 technologies to unravel and engineer these complex regulatory networks for targeted overproduction of valuable phytochemicals.

## Introduction

Plants synthesize a vast array of natural products, which can be broadly categorized as primary and secondary metabolites. Primary metabolites, such as nucleotides, amino acids, and acyl lipids, are indispensable for the fundamental growth and development of all plant species. In contrast, secondary metabolites are typically species-specific and fulfill vital functions in plant development, stress adaptation, and ecological interactions [1]. Based on their chemical structures and biosynthetic origins, these compounds are classified into three major classes: terpenoids, phenolic compounds, and alkaloids. They are not only important sources for pharmaceuticals, nutraceuticals, fragrances, and cosmetics but also possess immense socioeconomic value, thereby garnering considerable research attention [2]. The biosynthesis of plant secondary metabolites is an intricate process subject to precise, multi-level regulation, in which transcription factors (TFs) serve a pivotal role [3]. Among the numerous TF families, the AP2/ERF (APETALA2/ethylene responsive factor) family constitutes a large, plant-specific class of regulatory proteins. They function as key molecular switches that integrate signals from endogenous developmental cues (e.g. phytohormones) and exogenous environmental stimuli (e.g. biotic and abiotic stresses), translating these inputs into the precise regulation of downstream gene expression.

**Figure 1:**
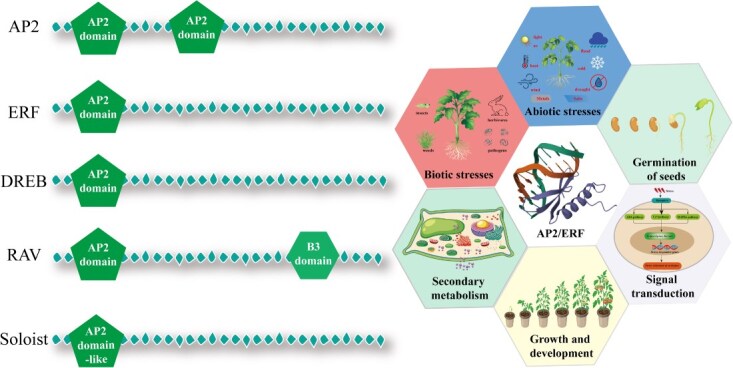
Classification and functional modules of AP2/ERF transcription factors. The AP2/ERF superfamily is categorized into five subfamilies—AP2, ERF, DREB, RAV, and Soloist—based on the number and type of conserved domains they contain. The AP2 subfamily is characterized by the presence of two repeated AP2 domains, whereas the ERF and DREB subfamilies each contain a single AP2 domain. The RAV subfamily consists of one AP2 domain combined with an additional B3 domain, and the Soloist subfamily features a more divergent AP2-like domain. These transcription factors play regulatory roles in multiple interconnected physiological processes in plants, which can be conceptualized as overlapping ‘functional modules’. These modules encompass responses to both biotic (e.g. pathogens, insects) and abiotic stresses (e.g. drought, cold, salinity), seed germination, signal transduction, growth and development, as well as the biosynthesis of secondary metabolites.

Members of the AP2/ERF superfamily all contain one or more conserved AP2/ERF DNA-binding domains, which are characterized by two conserved elements: YRG and RAYD. Based on the number of AP2/ERF domains and the presence of other domains, this superfamily is classified into five subfamilies: AP2, ERF, DREB, RAV, and Soloist. This structural diversity directly underlies their functional specificity (Fig. 1): different subfamily members activate or repress specific metabolic pathways by recognizing distinct cis-acting elements in the promoters of their downstream target genes. For instance, the ERF subfamily preferentially binds to the GCC-box, whereas the DREB subfamily recognizes DRE/CRT elements (dehydration-responsive element/C-repeat).

The AP2/ERF TFs are named based on their ability to respond to ethylene regulation. However, this function is not universally shared across the AP2/ERF family, nor is its conserved domain directly influenced by ethylene signaling. Despite this, the nomenclature has persisted to the present day [4]. The AP2/ERF TFs family is classified into five subfamilies (AP2, ERF, DREB, RAV, and Soloist) based on the number of AP2/ERF domains and the presence of additional structural domains [5]. The AP2 subfamily contains two repeated AP2/ERF domains, while both ERF and DREB subfamilies possess only one AP2/ERF domain (Fig. 1). The RAV family is characterized by containing one AP2/ERF domain along with an additional B3 domain, whereas the Soloist family also contains a single AP2/ERF domain but exhibits significant structural divergence from other subfamilies and maintains highly conserved nucleotide sequences across most plant species. AP2/ERF domains contain one α-helix and three β-sheet regions. Additionally, the DREB subfamily can be further divided into six subgroups: A1–A6, while the ERF subfamily comprises six subgroups: B1–B6. These TFs are capable of recognizing and binding to a GCC-box or other reported cis-elements (such as CE1, DRE/CRT) sequence within the promoters of target genes, thereby regulating their expression. AP2/ERF TFs have been implicated in a variety of signaling pathways. Beyond their involvement in secondary metabolite biosynthesis, AP2/ERF TFs have been confirmed to be pivotal in numerous plant processes, such as defense responses, growth and development. The roles of AP2/ERF TFs are summarized in the right part of Fig. 1.

**Figure 2:**
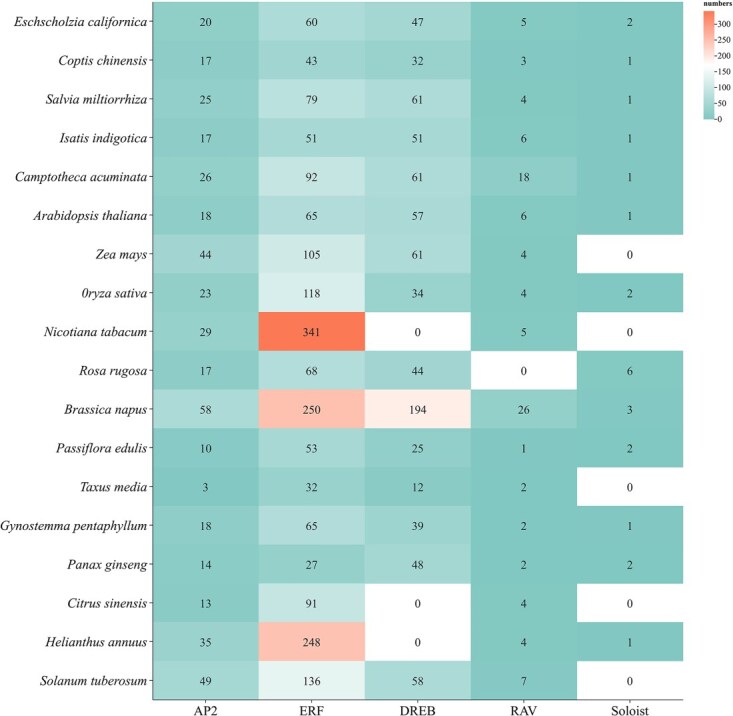
The current numbers of AP2, ERF, DREB RAV, and Soloist among the various species that have dominated.

The number of AP2/ERF TFs and their subfamily members varies among different plant species with Fig. 2. Analysis revealed that AP2/ERF TFs are abundant in these plants, with potato harboring as many as 246 AP2/ERF members. In currently reported plants, ERF and DREB subfamilies collectively account for over 50% of the total AP2/ERF family [6]. However, the Soloist subfamily is rare, with most plants containing only one member, while no Soloist AP2/ERF TFs have been identified in rice [7]. With the development of omics technology, AP2/ERF TFS of more and more species have been identified. The total number of identified genes per species ranged substantially, from a minimum of 49 in *Taxus* media to a maximum of 531 in *Brassica napu*s. The ERF subfamily consistently represented the largest group in most species, notably comprising 341 members in *Nicotiana tabacum* (constituting 91% of its total AP2/ERF genes). Significant interspecific variation was observed: the DREB subfamily was absent (*Nicotiana tabacum*, *Citrus sinensis*, *Helianthus annuus*) or markedly reduced (*Oryza sativa*, *n* = 34) in several species, while exceptionally abundant in others (*Brassica napus*, *n* = 194). The RAV and Soloist subfamilies were generally the smallest, with RAV counts ranging from 0 (*Rosa rugosa*) to 26 (*Brassica napus*, *Camptotheca acuminata*) and Soloist counts typically between 0 and 2, except in *Rosa rugosa* (*n* = 6).

This review systematically summarizes the current understanding of the molecular mechanisms by which AP2/ERF transcription factors regulate the biosynthesis of the three major classes of plant secondary metabolites. We will first outline the primary modes of AP2/ERF-mediated regulation—direct, indirect, co-regulation, and self-regulation. Subsequently, we will delve into specific case studies to illustrate how these TFs control the production of valuable terpenoids, phenolic compounds, and alkaloids. Our analysis encompasses approximately 40 phylogenetically diverse plant species where AP2/ERF TFs have been experimentally validated to regulate these compound categories, as systematically cataloged in Fig. S1. The classification of each species is based on the primary metabolite class associated with AP2/ERF regulation in the cited literature. By elucidating these complex regulatory networks, this review aims to provide a comprehensive framework for future research and for the targeted metabolic engineering of high-value phytochemicals in plants.

## Molecular mechanisms of AP2/ERF TFs in the regulation of plant secondary metabolites biosynthesis

**Figure 3:**
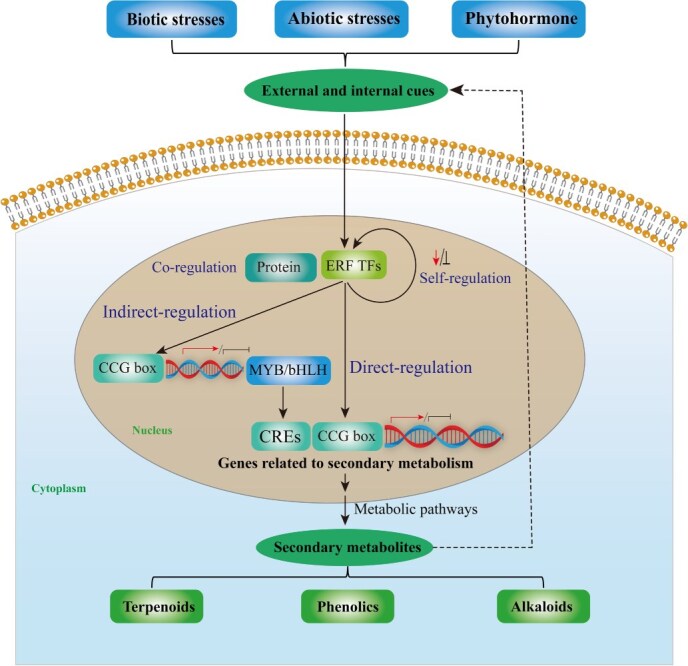
Schematic of molecular mechanisms by which AP2/ERF TFs regulate secondary metabolite biosynthesis. AP2/ERF TFs integrate external signals (biotic/abiotic stresses) and phytohormones to orchestrate metabolic pathways. The regulatory mechanisms are classified as: (i) Direct-regulation, where AP2/ERF TFs bind to CREs, such as the GCC-box, CE1 and DRE/CRT, in the promoters of biosynthetic genes (e.g. acyclic diterpene synthase (ADS) for artemisinin); (ii) Indirect-regulation, Refers to the regulation of upstream signaling components or other transcription factors; (iii) Co-regulation, AP2/ERF TFs form transcriptional complexes with other proteins (such as MYB and bHLH) to collectively regulate target genes; and (iv) self-regulation, involving transcriptional autoregulation and post-translational modifications, which fine-tune TF activity. The ultimate outputs are the biosynthesis of major metabolite classes, including alkaloids, terpenoids, and phenolics.

The TFs primarily regulate gene expression by binding cis-regulatory elements (CREs) in target gene promoters. The characteristic CRE recognized by AP2/ERF TFs is the GCC-box or CE1, DRE/CRT. Each AP2/ERF TF binds to this GCC-box within the promoters of specific downstream gene sets, modulating their expression. Furthermore, the AP2/ERF transcription factor can form complexes with various proteins, thereby enabling precise regulation of downstream genes. Consequently, during secondary metabolite biosynthesis, AP2/ERF transcription factors display diverse regulatory patterns, which are primarily determined by their target genes and interacting proteins [8]. These TFs can respond to plant hormones as well as biotic and abiotic stress signals, integrating endogenous developmental cues with exogenous environmental stimuli to precisely regulate gene expression. This ensures normal plant growth and adaptive responses [9]. In this review, the regulatory mechanisms of AP2/ERF TFs in secondary metabolism are classified into four types: direct regulation, indirect regulation, synergistic regulation, and self-regulation as illustrated by the regulatory network in Fig. 3.

### Direct transcriptional regulation of biosynthetic genes

AP2/ERF TFs directly bind to specific cis-acting elements in the promoters of secondary metabolite biosynthetic genes, activating or repressing their transcription.AP2/ERF TFs recognize conserved DNA motifs, such as GCC-box (AGCCGCC), CE1 and DRE/CRT elements, in the promoters of target genes. AaERF1 in *A. annua* activates the promoter of *ADS*, a key enzyme in artemisinin biosynthesis, by binding to GCC-box CE1 and DRE/CRT elements. Direct regulation allows rapid and precise control of metabolic pathways, enabling plants to dynamically adjust secondary metabolite production in response to developmental or environmental cues.

### Indirect regulation: control via transcriptional cascades and signaling hubs

Indirect regulation describes a mechanism where AP2/ERF transcription factors modulate secondary metabolism without directly binding to the promoters of the final biosynthetic genes, instead playing an ‘upstream’ or ‘relay’ role in the regulatory network. This mode can be achieved by initiating a transcriptional cascade, where an AP2/ERF factor activates an intermediary transcription factor that, in turn, directly controls the pathway genes. A well-documented example is found in *N. benthamiana*, where NtERF221 indirectly promotes nicotine biosynthesis by upregulating the expression of the bHLH transcription factor NtMYC2, which subsequently activates the key biosynthetic genes [10]. Alternatively, AP2/ERF TFs can function as central signaling hubs that integrate upstream phytohormone signals. In *Arabidopsis*, for example, ERF1 acts as a crucial integrator of jasmonate (JA) and ethylene (ET) signaling, indirectly promoting the synthesis of defense-related glucosinolates by activating a broad set of downstream stress-responsive genes rather than the biosynthetic enzymes themselves. Through these indirect approaches, AP2/ERF TFs are able to link broad stress and developmental signals to specific metabolic pathways, enabling a higher tier of resource allocation and regulatory control.

### Transcriptional co-regulation of biosynthetic genes

AP2/ERF TFs coordinate with other regulators through co-regulation to fine-tune plant secondary metabolite biosynthesis, often synergizing or competing with partner proteins to achieve precise control. For example, they form heterodimers or multiprotein complexes to amplify transcriptional activation: in *C. roseus*, CrERF5 interacts with bHLH TFs to co-activate terpenoid indole alkaloid (TIA) biosynthetic genes, enhancing vincristine and vinblastine production [11]. Similarly, AP2/ERF TFs may bind adjacent promoter regions with MYB or WRKY factors, creating a synergistic effect; In *P. ussuriensis*, PuERF27 partners with PuMYB10 to jointly activate the promoters of anthocyanins pathway genes [12]. In anthocyanin biosynthesis, AP2/ERF factors act as crucial partners of the MYB-bHLH-WD40 (MBW) complex. They can synergistically enhance the activation of target genes like PpUFGT by stabilizing the complex or, conversely, competitively bind to the bHLH partner to repress transcription, thereby fine-tuning pigment production. Similarly, AP2/ERF TFs form heteromeric complexes with WRKY TFs to co-regulate pathways involved in tannin metabolism and defense responses. This co-regulation is also evident in terpenoid indole alkaloid (TIA) synthesis, where factors like CrERF5 interact with bHLH proteins to activate pathway genes. These cooperative and antagonistic interactions allow AP2/ERF TFs to integrate diverse signals and exert highly specific spatiotemporal control over metabolic networks. Conversely, competitive co-regulation can suppress pathways when AP2/ERF TFs antagonize other regulators by occupying shared DNA-binding sites or sequestering co-activators. This cooperative or antagonistic interplay ensures spatiotemporal specificity and metabolic flexibility, balancing resource allocation between growth and defense while avoiding overaccumulation of toxic intermediates. Such co-regulatory networks highlight the adaptability of AP2/ERF TFs in integrating diverse signals to optimize secondary metabolite production under varying environmental or developmental conditions.

### Self-regulatory mechanisms: maintaining homeostasis of the regulators

To ensure that metabolic responses are proportional and transient, the activity and abundance of AP2/ERF transcription factors are themselves subject to tight control. We distinguish this from systemic feedback by focusing on mechanisms that directly modulate the TF itself, which can be categorized into two precise modes:

#### Transcriptional autoregulation

This mechanism involves a feedback loop where an AP2/ERF factor directly or indirectly controls the transcription of its own gene. This is often a negative feedback loop designed to prevent runaway activation. In *Arabidopsis*, AtERF1, upon activation by stress signals, initiates a cascade that ultimately leads to the suppression of its own expression [13]. This autoregulatory circuit ensures that the defense response, including the accumulation of metabolites like camalexin, is attenuated once the initial stress has been addressed, thereby conserving cellular resources.

**Figure 4:**
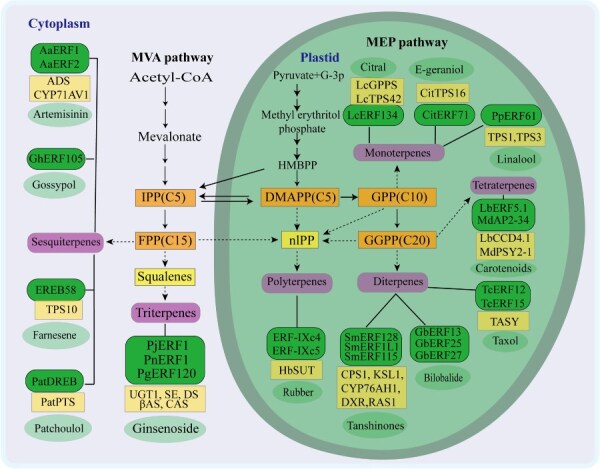
Metabolic pathways of terpenes and the related AP2/ERF TFs. This figure illustrates the two core biosynthetic pathways for terpenoids in plants: the MVA pathway in the cytoplasm and the MEP pathway in the plastids. These pathways produce the universal C5 precursors, IPP and DMAPP. Subsequent condensation reactions form geranyl diphosphate (GPP, C10), farnesyl diphosphate (FPP, C15), and geranylgeranyl diphosphate (GGPP, C20), which are the skeletons for monoterpenes, sesquiterpenes, diterpenes, triterpenes, tetraterpenes, and polyterpenes. The green boxes indicate specific AP2/ERF transcription factors that have been experimentally shown to regulate the biosynthesis of the corresponding compounds (e.g., Artemisinin, Ginsenoside, Taxol).

#### Regulation by post-translational modification

This mode involves the modification of the AP2/ERF protein itself, which rapidly alters its stability, activity, or subcellular localization without changing its gene expression level. A prime example is the regulation of NtERF172 in *N. tabacum*. The NtERF172 protein undergoes phosphorylation-dependent degradation, a process that limits the jasmonate-mediated activation of nicotine biosynthesis. This mechanism allows the cell to dynamically adjust the concentration of active NtERF172 protein in direct proportion to the intensity and duration of the stress signal, ensuring a precisely calibrated metabolic output [14].

## Regulation of terpenoids biosynthesis by AP2/ERF TFs

Terpenoids, representing the most structurally diverse class of plant secondary metabolites, are ubiquitously distributed across natural systems. To date, over 80 000 distinct terpenoid compounds have been characterized, a testament to their remarkable chemical versatility. Functionally diversified, these metabolites fulfill dual biological and practical roles: they are indispensable for plant growth, developmental processes, and ecological defense mechanisms, while simultaneously serving as invaluable resources for pharmaceutical development (e.g. artemisinin and taxol), industrial applications (biofuels, rubber), and consumer products (fragrances, flavorings). Structural classification of terpenoids follows their isoprene unit composition, encompassing: hemiterpenes, monoterpenes, sesquiterpenes, diterpenes, triterpenes and polyterpenes [15]. The biosynthetic pathway of terpenoid compounds is shown in Fig. 4: isopentenyl diphosphate (IPP) and dimethylallyl diphosphate (DMAPP). These isoprenoid building blocks are generated through compartmentalized metabolic pathway–the mevalonate (MVA) pathway in the cytoplasm and the methylerythritol phosphate (MEP) pathway in plastids. Notably, terpenoids with high demand, including artemisinin, taxol, and ginsenosides, have emerged as research hotspots in recent years [16]. At present, the role of AP2/ERF TFs in regulating the synthesis of these substances is continuously being confirmed and further explored.

### Regulation of monoterpene biosynthesis

Monoterpenes, prominent constituents in essential oils of aromatic and medicinal plants, constitute a significant class of plant secondary metabolites. Valued industrially as flavor additives in food, beverages, and perfumes [17], they also exhibit diverse biological activities including anti-inflammatory, antibacterial, and antioxidant effects. Their potential as medicinal agents, particularly demonstrated anticancer activity in vitro has attracted substantial research interest [18]. Molecular studies reveal direct AP2/ERF TFs regulation of biosynthesis, LcERF134 activates monoterpene production in *Litsea cubeba* by binding GCC-box elements in the *LcTPS42* and *LcGPPS-SSU1* promoters. Transient overexpression of LcERF134 significantly enhanced monoterpene accumulation and key pathway gene expression [19]. Similarly, sweet orange (*Citrus sinensis* Osbeck) flavor contributor E-geraniol is synthesized by CitTPS16. CitERF71 promotes its production by binding ACCCGCC and GGCGGG motifs in the *CitTPS16* promoter. In peach, linalool biosynthesis by *PpTPS1* and *PpTPS3* is activated by PpERF61 through DRE/CRT motif binding. Transient overexpression of *PpERF61* increased both terpene synthase expression and linalool content [20].

### Sesquiterpenes

Sesquiterpenes (C15 terpenoids derived from three isoprene units) are secondary metabolites with significant medicinal potential, particularly in cancer [21]. Occurring in higher plants, fungi, and marine organisms，they exist as hydrocarbons or oxygenated derivatives like alcohols, ketones, acids, aldehydes, and lactones [22]. These compounds exhibit cytotoxic effects against diverse carcinomas (e.g. prostate, breast, colon, leukemia, lung, liver), with notable specificity against prostate cancer; several have advanced to human clinical trials [23].

Artemisinin is a sesquiterpene lactone derived from the herb *Artemisia annua* L. Artemisinin possesses significant medicinal properties and plays a vital role in malaria treatment worldwide [24]. The ADS and cytochrome P450 monooxygenase 71AV1 (CYP71AV1) are two key genes involved in the artemisinin biosynthesis pathway. AaERF1 and AaERF2 can activate the expression of *ADS* and CYP71AV1 genes by binding to their upstream promoters. Overexpression of *AaERF1* and *AaERF2* in *A. annua* significantly enhances artemisinin content. In contrast, RNA interference (RNAi) lines of *A. annua* exhibit reduced production of artemisinin. Another trichome-specific AP2/ERF TF, AaORA, has been shown to positively regulate the accumulation of artemisinin and dihydroartemisinic acid in *A. annua*. In *A. annua*, overexpression and RNA interference (RNAi) of *AaORA* significantly affected the expression levels of key genes including *ADS*, *CYP71AV1*, *DBR2* and *AaERF1*. The modulation of these genes’ expression, whether up-regulated or down-regulated, led to a corresponding significant increase or decrease in the levels of artemisinin and dihydroartemisinic acid [25]. Additionally, the AP2 TF TAR1 promotes trichome development and artemisinin biosynthesis. TAR1 silencing altered trichome morphology/wax composition and reduced artemisinin, while overexpression increased it. Silencing/overexpressing TAR1 altered key artemisinin pathway genes, with EMSA, Y1H, and GUS assays indicating *ADS* and *CYP71AV1* as TAR1 direct targets [26].

### Regulation of diterpenes biosynthesis

Diterpenes are a class of natural compounds characterized by a 20-carbon skeleton derived from four isoprene units. These compounds have been extensively applied in pharmaceutical development, food additives, and cosmetics [27]. Notably, plant-derived diterpenoids have attracted significant attention in drug discovery due to their diverse pharmacological activities, with several diterpenoid-based therapeutics already in clinical use [28]. Representative examples include *Salvia miltiorrhiza*-based formulations such as Compound Danshen Dripping Pills/Tablets (containing tanshinone IIA) for preventing or treating coronary heart disease, stroke, and arthritis; Paclitaxel Injection (containing taxol from *Taxus brevifolia*) for ovarian, pancreatic, and breast cancers; Ginkgolide B Injection (derived from Ginkgo biloba) for ischemic stroke therapy; and Tripterygium Tablets (containing triptolide from *Tripterygium wilfordii*) for rheumatoid arthritis management. These clinical applications underscore the substantial medicinal value of plant-derived diterpenoids. Furthermore, research on the regulatory mechanisms of AP2/ERF TFs (TFs) in diterpenoid biosynthesis has revealed diverse roles, encompassing both positive and negative regulation, as well as direct transcriptional activation and indirect pathway modulation, with ongoing studies continually expanding our understanding of these complex networks.

*Salvia miltiorrhiza* is a well-known traditional Chinese medicine widely used in the treatment of cardiovascular diseases. The active ingredients of *S. miltiorrhiza* can be divided into two categories: hydrophilic compounds and lipophilic compounds [29]. Although tanshinones and salvianolic acids have important clinical application value, the fact that these compounds are only produced by *S. miltiorrhiza* at low concentrations restricts the development and application of *S. miltiorrhiza*. Tanshinones are synthesized from IPP and DMAPP through two different pathways, the methylerythritol MEP and the MVA [30]. There are two pathways for the production of salvianolic acids, one is the tyrosine-derived pathway and the other is the phenylpropanoid pathway. In the tyrosine derivatization pathway, l-tyrosine is synthesized into 4-hydroxyphenyllactic acid (4HPLA) by tyrosine aminotransferase (TAT) and 4-hydroxyphenylpyruvate reductase (HPR), and then 3,4-dihydroxyphenyllactic acid is generated. In the phenylpropanoid pathway, l-phenylalanine is converted into 4-coumaroyl-CoA ligase (4CL) by phenylalanine ammonia lyase (PAL) and cinnamate 4-hydroxylase (C4H), and then rosmarinic acid synthase (RAS) and P450 monooxygenase couple 4HPLA and 4-coumaroyl-CoA to form salvianolic acid [31].

SmERF128 activate tanshinone biosynthesis by directly binding to GCC-box, CBF2, and RAA motifs in the promoters of key pathway genes (*SmCPS1*, *SmKSL1*, *SmCYP76AH1*) to activate their expression, with corresponding changes in tanshinone content observed in overexpression and silencing experiments [32]. SmERF115 acts as a positive regulator of salvianolic acid biosynthesis, directly binding to the SmRAS1 promoter (confirmed by Y1H, EMSA, and dual luciferase assays) and altering salvianolic acid/tanshinone levels and SmRAS1 expression in overexpression/silencing lines. Transcriptome analysis of overexpressing roots further validated upregulation of salvianolic acid synthesis genes [33]. SmERF1L1 increases tanshinone accumulation (while reducing salvianolic acid) by upregulating key tanshinone synthesis genes (*SmDXR*, *SmDXS2*, *SmHMGS*, *SmKSL*), with direct binding to the GCC box in the SmDXR promoter confirmed by multiple assays [34]. SmERF6 activate tanshinone biosynthesis by directly binding to the GCC box of the SmKSL1 and SmCPS1 promoters and activating their transcription. At the same time, the accumulation of tanshinone increased in hairy roots overexpressing *SmERF6*, while silencing *SmERF6* by RNAi led to a decrease in tanshinone content. In addition, the content determination results also showed that the accumulation of tanshinone maintained a dynamic balance with the total phenolic acid and flavonoid content in *S. miltiorrhiza*. SmERF8 promotes tanshinone biosynthesis by directly binding to the SmKSL1 promoter and activating its transcription, as evidenced by corresponding tanshinone content changes in overexpression and RNAi lines [35].

Paclitaxel is a natural secondary metabolite isolated and purified from the bark of the yew plant of the *Tasus*. It has good anti-tumor effects. Its demand is large and its wild resources are endangered, which makes the development and utilization of paclitaxel very difficult [36]. The paclitaxel used in the market mainly comes from chemical semi-synthesis. In the synthesis pathway of paclitaxel, the steps before the synthesis of geranylgeranyl pyrophosphate (GGPP) are similar to the synthesis pathway of artemisinin [37]. GGPP is used as a precursor to generate taxene under the catalysis of taxadiene synthase (TASY). Taxene is then synthesized into the A, B, and C rings of the taxene compound through a series of enzyme-catalyzed reactions, and then continuously oxidized by multi-step taxane hydroxylase, including taxene 5α-hydroxylase, taxenol 5α-acetyloxytransferase (T5αH), taxane 10β-hydroxylase (T10βH), taxane 7β-hydroxylase, taxane 2α-hydroxylase, taxane 13α-hydroxylase (T13αH), taxane 2α-benzyl acyltransferase, taxane 14β-hydroxylase, C13-phenylpropionic acid-side chain-CoA transferase, taxane C13-side chain-N-benzoyltransferase, 10-deacetylbaccatin III-10β-acetyltransferase, etc., to finally produce paclitaxel [38].

Jasmonic acid (JA) treatment of *Taxus cuspidata* suspension cells significantly enhances taxol accumulation. Yeast one-hybrid screening identified TcAP2, an AP2/ERF-family TFs (TF) with high homology to known regulators, suggesting its role in activating stress-responsive genes and modulating key enzyme-encoding genes (e.g. TASY, T10βH, T13αH) to promote taxol biosynthesis [39]. Furthermore, Dai *et al.* characterized another AP2-class TF, TcDREB, which binds to the GCC box—a methyl jasmonate (MeJA)-responsive cis-element—in the promoters of TASY, T10βH, T13αH, and T5αH, indicating its regulatory role in the isoprenoid metabolic pathway underlying taxol production. Two JA-responsive ERF transcription factors, TcERF12 and TcERF15, exhibit opposing roles in the regulation of taxol synthesis within Taxus chinensis. When TcERF12 is overexpressed, the taxadiene synthase gene's expression is suppressed, while the expression of this gene is enhanced by TcERF15 in *T. chinensis* cells.

### Regulation of triterpenes biosynthesis

Triterpenoids have a wide variety of species and sources, and exist in many medicinal plants. They exist in free form or in the form of glycosides or esters combined with sugars. Most of them have biological activities such as signaling molecules and stress response. In modern pharmacological studies, it has been found that it has anti-inflammatory, antibacterial, antiviral, anti-tumor, anti-fertility and immunomodulatory effects [40]. Triterpenoids are the most abundant and diverse natural products in plants, mainly in the form of tetracyclic triterpenoids and pentacyclic triterpenoids. They are composed of 2,3-oxidosqualene, oxidosqualene cyclase, and triterpenoids. OSC catalyzes cyclization to produce more than 100 different triterpene skeletons, resulting in a huge structural diversity [41].

Ginsenosides, the major bioactive triterpenoid saponins found in *Panax* species and *Gynostemma pentaphyllum*, exhibit diverse pharmacological properties, including antioxidant, anti-inflammatory, vasodilatory, anti-allergic, and anti-diabetic effects [42]. Studies demonstrate that AP2/ERF TFs regulate triterpenoid saponin biosynthesis by directly binding to the GCC-box cis-element in the promoters of target genes. PnERF1 (from *Panax notoginseng*) significantly enhances total saponin content in transgenic cell lines and specifically promotes the accumulation of monomeric ginsenosides, including Rg3, Rh1, Rd, Rg1, F1, and Re, confirming its role as a positive regulator [43]. Similarly, PjERF1 (from *Panax japonicus*) activates the promoters of key enzyme genes (*PjβAS*, *PjCAS*, and *PjSE*), upregulating triterpenoid biosynthetic pathway genes and substantially increasing the levels of chikusetsusaponin IV/IVa and ginsenosides (Rd, Rb1, Re, and R0) [44]. In contrast, overexpression of PgERF120 (from *Panax ginseng*) exhibits differential regulatory effects. It upregulates the glycosyltransferase gene PgUGT1, promoting the conversion of Rh2 and Rg3 into Rd, which is further metabolized to Rc, ultimately leading to reduced Rh2/Rg3 levels and increased Rd/Rc accumulation [45].

### Regulation of tetraterpenes and polyterpenes biosynthesis

Carotenoids are essential isoprenoids in plants, crucial for photosynthesis, photoprotection, and as precursors to apocarotenoids—hormones and signaling molecules vital for development and stress responses [46]. The biosynthetic pathway begins with phytoene synthase (PSY) converting geranylgeranyl diphosphate to 15-cis-phytoene. Subsequent catalysis by phytoene desaturase (PDS), 15-cis-ζ-carotene isomerase (Z-ISO), ζ-carotene desaturase (ZDS), and carotenoid isomerase (CRTISO) sequentially converts this precursor into all-trans-lycopene through a series of desaturation and isomerization steps [47]. AP2/ERF TFs critically regulate carotenoid metabolism; for example, apple MdAP2–34 enhances phytoene and β-carotene accumulation by directly activating the key gene *MdPSY2–1* promoter, though it minimally affects lutein [48]. Similarly, in *Lycium* (goji berry), the ERF family TFs LbERF5.1 activates the expression of LbCCD4.1 (carotenoid cleavage dioxygenase 4) by binding to its promoter, leading to the degradation of carotenoids, such as β-carotene and β-cryptoxanthin [49]. Concurrently, it regulates the differential expression of synthesis-related genes like *GGPPS* and *PSY*, forming a dynamic balance between synthesis and degradation. In the microalga *Dunaliella parva*, DpAP2, a conserved AP2/ERF TFs, enhances carotenoid accumulation by binding to target gene promoters and interacts with proteins involved in DNA binding, kinase activity, and metabolic regulation, establishing a multi-level regulatory module [50]. During papaya fruit ripening, CpEIN3a (a core ethylene signaling factor in the EIN3/EIL family) intersects with the AP2/ERF regulatory network. It directly binds to promoters of carotenoid biosynthesis genes, such as CpPDS4 and CpCHY-b and physically interacts with CpNAC2, to form a transcriptional complex that synergistically activates the carotenoid biosynthesis pathway. These studies reveal that the AP2/ERF family not only directly regulates synthesis-related enzymes but also modulates carotenoid metabolism through interactions with cleavage enzymes or other TFs (e.g. NAC).

Rubber is a polyterpene obtained from rubber trees (*Hevea brasiliensis* Muell) or *Taraxacum koksaghyz* Rodin, both of which are regarded as vital economic crops. However, due to the complex biosynthetic pathways and regulatory mechanisms of rubber, some challenges have been brought to the research on rubber synthesis [51]. HbERF-IXc4 and HbERF-IXc5 as key transcriptional regulators in rubber biosynthesis. HbERF-IXc4 directly activates the promoter of the sucrose transporter gene *HbSUT3*, thereby enhancing rubber yield [52]. Meanwhile, overexpression of *HbERF-IXc5* promotes plant growth, increases starch accumulation, and stimulates laticifer cell differentiation in response to abiotic stresses such as water deficit, cold, and salinity [53].

Terpenoids, with over 80 000 identified types, are the most structurally diverse secondary metabolites and play a crucial role in maintaining plant growth, development, and defense mechanisms. They are also important sources for pharmaceuticals (such as artemisinin and paclitaxel) and industrial raw materials (such as rubber and biofuels). The biosynthesis of terpenoids begins with the universal precursors IPP/DMAPP, which are generated through the cytosolic MVA MEP pathway. These precursors undergo condensation, cyclization, and other reactions to form various types of terpenoids. The AP2/ERF transcription factor family plays a crucial role in regulating terpenoid biosynthesis pathways by directly binding to cis-acting elements, such as GCC-box and DRE/CRT, in the promoters of target genes (Table 1). This regulation includes both direct transcriptional activation (for example, PjERF1 binding to the *PjβAS* promoter during ginsenoside synthesis) and modulation of metabolic homeostasis (such as PgERF120 regulating saponin components through glycosyltransferase *PgUGT1*, and LbERF5.1 balancing carotenoid levels by activating catabolic enzyme LbCCD4.1). These factors also respond to hormonal signals, such as jasmonate, to form complex regulatory networks. As core molecular switches for terpenoid synthesis, AP2/ERF transcription factors are critical targets for the artificial, directional production of high-value terpenoids.

**Table 1:** The list of reported AP2/ERF TFs involved in the regulation of terpenes biosynthesis

**Metabolite Class**	**Related compounds**	**Species**	**AP2/ERF TFs**	**Downstream genes**	**References**
**Monoterpenes**	Citral	*Litsea cubeba*	LcERF134	LcTPS42, LcGPPS, SSU1	[19]
	Linalool	*Prunus persica*	PpERF61	*PpTPS1*, *PpTPS3*	[20]
	E-geraniol	*Citrus sinensis*	CitERF71	*CitTPS16*	[54]
**Sesquiterpenes**	Artemisinin	*Artemisia annua*	AaERF1/2	ADS, CYP71AV1	[55]
			AaORA	ADS, CYP71AV1, DBR2	[25]
			TAR1	ADS, CYP71AV1	[26]
	Gossypol	*Gossypium hirsutum*	GhERF105	-	[56]
	Farnesene	*Zea mays*	EREB58	*TPS10*	[57]
	Patchoulol	*Pogostemon cablin*	PatDREB	*PatPTS*	[58]
**Diterpenes**	Taxol	Taxus chinensis	TcERF12/15	TASY	[59]
	Tanshinones	*Salvia miltiorrhiza*	SmERF128	CPS1, KSL1, CYP76AH1	[32]
			SmERF115	SmDXR	[33]
			SmERF1L1	SmRAS1	[34]
			SmERF6	SmKSL1, SmCPS1	[60]
			SmERF8	SmKSL1	[35]
	Bilobalide	*Ginkgo biloba*	GbERF13/25/27	-	[61]
**Triterpenes**	ginsenosides	*Panax notoginseng*	PnERF1	*PnSE,PnDS*	[43]
		*Panax ginseng*	PgERF120	*PgUGT1*	[45]
	chikusetsusaponin	*Panax japonicus*	PjERF1	*PjβAS*, *PjCAS*, *PjSE*	[44]
**Tetraterpenes**	Carotenoids	*Lycium barbarum*	LbERF5.1	*LbCCD4.1*	[49]
		*Malus domestica*	MdAP2–34	*MdPSY2–1*	[48]
		*Dunaliella parva*	DpAP2	PDS, GGPS	[50]
**Polyterpenes**	Rubber	*Hevea brasiliensis*	HbERF-IXc4/5	HbSUT	[52]

- indicates that the downstream regulatory genes have not been reported.

AP2/ERF transcription factors play a pivotal role in regulating the biosynthesis of plant terpenoids. They precisely control the metabolic flux from the MVA and MEP pathways by directly binding to cis-acting elements, such as the GCC-box or DRE/CRT elements, within the promoters of key enzyme-encoding genes (e.g. *ADS*, *TPS*, *KSL*). This regulatory network is deeply integrated with hormonal signals, notably JA, enabling plants to dynamically adjust the production of terpenoids like artemisinin, tanshinone, and paclitaxel in response to internal and external environmental cues. Table 1 specifically presents the AP2/ERF TFs in different plants that regulate various terpenoid compounds and the specific target genes they act upon. In contrast to terpenoids, which derive from isoprene precursors, another major class of plant secondary metabolites—phenolic compounds—is synthesized primarily through the shikimate and phenylpropanoid pathways. The subsequent section will explore how this versatile family of transcription factors, AP2/ERF, employs analogous regulatory logic (e.g. integration of hormonal signals, direct regulation of target genes) to orchestrate this fundamentally different metabolic network and govern the biosynthesis of important substances such as flavonoids, lignin, and anthocyanins.

## Regulation of phenolic compounds biosynthesis by AP2/ERF TFs

**Figure 5:**
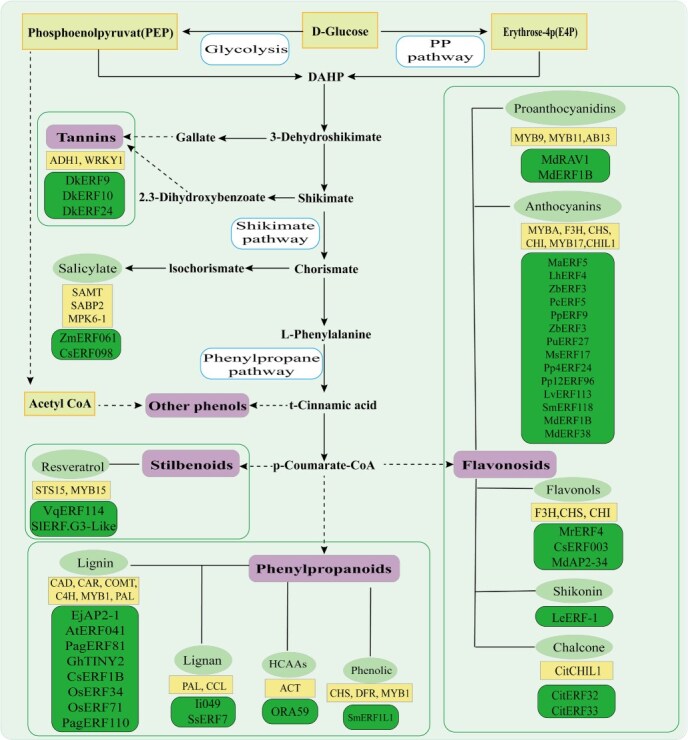
Phenolic compound metabolic pathways and the involved AP2/ERF TFs. This figure outlines the major biosynthetic routes for phenolic compounds in plants, originating from primary metabolism (Glycolysis and Pentose Phosphate pathway) and proceeding through the core Shikimate and Phenylpropane pathways. The key intermediate, L-Phenylalanine, serves as the precursor for a wide array of phenolic substances. The diagram shows major subclasses derived from these pathways, including flavonoids (e.g., anthocyanins, flavonols), lignin, stilbenoids (e.g., resveratrol), and tannins. The green boxes list the names of AP2/ERF transcription factors that have been functionally characterized to regulate the biosynthesis of the corresponding phenolic subclass.

Phenolic compounds constitute a major class of plant-derived substances, distinguished by their hydroxylated aromatic carbon rings. They serve essential functions in plants, including providing structural support and aiding in stress responses, while also contributing numerous medicinal properties for the prevention and treatment of various human ailments [62]. The classification of phenolic compounds and their biosynthetic pathways are shown in Fig. 4: phenylpropanoids, flavonoids, stilbenes, tannins, and phenolic acids. Notably, phenylpropanoids can be further subdivided into distinct subclasses, including simple phenylpropanoids (e.g. phenylpropionic acids and phenylpropanols), coumarins, flavonoids, lignans, and lignins. Notably, flavonoids display an impressive array of over 10 000 unique species, which can be classified into types such as flavones, flavonols, flavanols, anthocyanidins, chalcones, among others [63]. According to reports, AP2/ERF TFs play a critical role in regulating genes that are involved in phenolic compounds biosynthesis pathways are shown in Fig. 5. By influencing the expression of phenolic compounds biosynthesis genes, AP2/ERF TFs significantly impact the biosynthesis processes of these important compounds, which in turn can affect plant growth, development, and overall health.

### Regulation of phenylpropanoids biosynthesis

AP2/ERF TFs regulate the phenylpropanoid metabolic pathway through direct or indirect mechanisms, exhibiting diverse biological functions across plant species. In *Populus simonii*, overexpression of the ERF76 gene significantly upregulates genes in the phenylpropanoid biosynthesis pathway, enhancing abiotic stress tolerance by coordinating phenylalanine metabolism and hormone signaling. In *Nicotiana tabacum*, NtERF13a directly binds to GCC/DRE elements in the promoters of key phenylpropanoid pathway genes (*NtHCT*, *NtF3′H*, and *NtANS*) [64], promoting the biosynthesis of chlorogenic acid, flavonoids, and lignin while improving salt and drought resistance. The moss-specific PpERF24 in *Physcomitrium* patens activates phenylpropanoid and jasmonate pathways to bolster fungal pathogen defense [65]. In tomato (*Solanum lycopersicum*), SlERF.G3-Like, directly activated by the ripening regulator SlRIN, coordinates fruit maturation with flavonoid metabolism, achieving a ~70% increase in resveratrol derivatives through genetic engineering [66]. Conversely, in Petunia, PhERF6 negatively regulates floral volatile biosynthesis by competitively binding to the DNA-binding domains of the EOBI protein, with its silencing markedly enhancing volatile emission. These studies highlight the bidirectional regulatory capacity of ERF family members in phenylpropanoid metabolism: positive regulation typically involves direct activation of metabolic enzyme genes or integration with stress signaling pathways to enhance stress resilience and secondary metabolite accumulation, while negative regulation occurs via interference with other TFs DNA-binding activity. These mechanisms suggest species and organ specific roles, offering critical targets for improving plant stress tolerance and metabolic engineering applications.

### Regulation of anthocyanin biosynthesis

Anthocyanins, as a kind of widely distributed, diverse, safe, and non-toxic flavonoids, can be used as pigments, but also a good antioxidant, and have a greater therapeutic effect in health care products [67]. Anthocyanin has antioxidant and anti-mutagenic properties, and is widely used in medicine, cosmetics, food and other industries. Anthocyanin biosynthesis is an important branch of plant secondary metabolism, and the process of synthesis is basically the same, although the types of anthocyanins and their accumulation patterns differ in different species [68]. The plant kingdom produces relatively few types of anthocyanins, with only slightly over 20 known to date [69]. Glycosylation is the most common modification, where anthocyanins form anthocyanidins by bonding with one or more glucose, rhamnose, arabinose, or other sugars via glycosidic linkages. The anthocyanin biosynthetic pathway represents one of the most extensively characterized secondary metabolic pathways in plants. In recent years, a majority of the enzymes and genes implicated in this biosynthetic pathway have been systematically identified and functionally validated. In plants, anthocyanins are derived from flavanones such as naringenin and eriodictyol, which themselves originate from the phenylpropanoid pathway. The biosynthesis primarily involves three steps: (i) the initial step from tyrosine to p-coumaroyl-CoA, (ii) the early step from p-coumaroyl-CoA and malonyl-CoA to dihydroquercetin, and (iii) the late step from dihydroquercetin to anthocyanidins. AP2/ERF TFs play a critical regulatory role in the anthocyanin biosynthetic pathway.

AP2/ERF TFs play a crucial role in the regulation of flavonoid secondary metabolism. Pp4ERF24 and Pp12ERF96 interact with PpMYB114, promote the binding of PpMYB114 to PpbHLH3, enhance the activation of PpMYB114 on the key gene PpUFGT for anthocyanin synthesis, and significantly induce anthocyanin accumulation [70]. Transient overexpression of *PuERF27* was found to activate genes expression of anthocyanin biosynthesis and enhance the accumulation of anthocyanins. In contrast, silencing of *PuERF27* resulted in a repression of anthocyanin accumulation. Y1H, EMSA, and GUS assays demonstrated that PuERF27 directly bind to and positively activate the promoters of *PuMYB10* and *PuGSTF12*. Furthermore, PuERF27 could conjunction with PuMYB10, synergistically transactivates the promoter of PuGSTF12 [12]. AP2/ERF TFs have also been shown to be involved in the regulation of anthocyanin biosynthesis in other plants, such as MaERF5 of *Morus alba* [71]; MdERF38 of *Malus domestica* [72]; MsERF17 of *Malus spectabilis* [73]; LvERF113 of *Lilium viviana* [74]; PcERF5 of *Pyrus communis* [75]; SmERF118 of *Solanum melongena* [76]; ZbERF3 of *Zanthoxylum bungeanum* [77]; MdERF109, MdERF1B, and MdERF38 of *Malus domestica* also positively activate anthocyanin biosynthesis [78]. AP2/ERF TFs can also inhibit anthocyanin synthesis. *Arabidopsis* AtERF4 and AtERF8 act as transcriptional repressors, reducing the production rate and accumulation of anthocyanins [79]. PpERF9 and PpERF105 can induce the expression of the inhibitory R2R3-MYB gene PpMYB140. It also competes with PpMYB10 and PpMYB114 TFs that promote anthocyanin biosynthesis to bind to bHLH3, forming a MYB140-bHLH-WDR complex to further inhibit anthocyanin biosynthesis [80]. In addition, LhERF4 in *Lilium* spp. and PpERF9 in *Pyrus pyrifolia* also inhibit anthocyanin biosynthesis [81].

### Regulation of carotenoids biosynthesis

Carotenoids, serving as one of the key pigment components responsible for imparting color in plants, play a pivotal role in both plant photosynthesis and human health. They are the principal source of vitamin A in the human body and exhibit a range of physiological functions, including antioxidant, immunomodulatory, anticancer, and anti-aging effects [82]. The biosynthetic pathway of carotenoids has been basically clarified, from GGPP to octahydro lycopene, which is the first carotenoid, and then converted to other carotenoids through dehydrogenation, cyclization, hydroxylation, epoxidation, etc. [83]. AP2/ERF TFs can be involved in the biosynthesis of carotenoids by activating multiple genes in the metabolic pathway. In Arabidopsis, AP2/ERF TFs increase carotenoid content by binding to the promoter of the octahydro lycopene synthase gene (PSY, phytoene synthase). Apple MdAP2–34 directly binds to and activates the functional carotenoid metabolism gene MdPSY2–1 [48]. Grapefruit (*Citrus maxima* (Burm) Merr.) CmERF23 affects the expression of carotenoid synthase genes, such as LYCE, LYCB2, and NCED2, and thus regulates carotenoid accumulation [84]. Similarly, CsERF061 (*Citrus sinensis*), SlPti4 (*Solanum lycopersicum*), and PpeERF2 and PpeERF3 (Prunus persica) TFs positively regulate carotenoid synthesis. In addition to positive regulation, tomato SlERF6 inhibited carotenoid biosynthesis by negatively regulating the expression of HSP21 and 1-deoxy-d-xylulose5-phosphate synthase (DXS, 1-deoxy-dxylulose5-phosphate synthase). In addition, the tomato AP2/ERF gene SlAP2a was shown to be a negative regulator of fruit ripening, exerting a wide range of regulatory effects on fleshy fruit ripening, mainly through negative regulation of the ethylene signaling pathway and key genes for carotenoid biosynthesis.

### Lignin

Lignin, a critical structural polymer in the plant secondary cell wall, provides mechanical rigidity and resistance to biotic and abiotic stress. Its biosynthesis branches from the phenylpropanoid pathway, initiating with the conversion of phenylalanine to cinnamic acid by phenylalanine ammonia-lyase (PAL). A cascade of enzymatic reactions catalyzed sequentially by cinnamate-4-hydroxylase (C4H), 4-coumarate-CoA ligase (4CL), coumarate-3-hydroxylase (C3H), caffeoyl-CoA O-methyltransferase (CCoAOMT), and cinnamoyl-CoA reductase (CCR) produces coniferaldehyde. This intermediate is then reduced by cinnamyl alcohol dehydrogenase (CAD) to yield monolignols (e.g. coniferyl alcohol), which are subsequently polymerized by enzymes such as dirigent protein (DIR) and pinoresinol reductase (PLR).

AP2/ERF TFs are closely associated with lignin biosynthesis in *Isatis indigotica*. They regulate lignin synthesis through two pathways: activating the salicylic acid (SA) signaling pathway and modulating key structural genes in the lignin metabolic pathway. In poplar (*Populus alba*), *PagERF81*—highly expressed in developing xylem—directly represses lignin biosynthesis genes (*PagCCR1*, *PagCAD6*, *Pag4CLL9*) by binding their GCC-box promoters. PagERF81 mutants displayed thicker walls with more lignin, smaller vessels, and longer fibers, while overexpression reduced lignin. Altered cell morphology likely resulted from disrupted lignin deposition rather than direct regulation of differentiation genes [85]. Similarly, *PagERF110* overexpression decreased secondary xylem deposition, reducing cellulose, xylose, and lignin content. It directly activated PagXND1d, a key *NAC* gene suppressing secondary wall formation, confirmed by Y1H, luciferase, and ChIP assays [86]. CsERF1B modulates lignin synthesis and JA/SA pathways in citrus fruit, promoting pericarp lignin accumulation to enhance disease resistance [87]. Overexpression of the AP2/ERF TFs *Ii049* in *Isatis indigotica* hairy roots leads to high expression levels of lignan/lignin biosynthesis genes, resulting in significant accumulation of lignans/lignin. Conversely, knocking out the Ii049 gene reduces the transcriptional levels of these genes, demonstrating that Ii049 positively activates lignan biosynthesis [88]. In rice, OsERF34 regulates the accumulation of cellulose and lignin, thereby promoting secondary cell wall thickening and strength in the lower internodes of the panicle [89]. OsERF71 was identified as a direct binder to the promoter of Oscinnamoyl-coenzyme A reductase 1, which is a crucial gene involved in lignin biosynthesis. Overexpression of *OsERF71* resulted in increased transcript levels of *OsCCR1*, *OsCCR10*, *OsCAD*, and *OsC4H* [90]. In sweet potato (*Ipomoea batatas* (L.) Lam.), the AP2/ERF TFs IbRAP2.4 upregulates the expression of structural genes in the lignin biosynthesis pathway, promoting the formation of transgenic sweet potato storage roots while inhibiting tuber expansion [13]. In loquat (*Eriobotrya japonica* (Thunb.) Lindl.), EjERF39 interacts with the lignin synthesis activator EjMYB8 to form a protein complex, synergistically enhancing the activation of the lignin biosynthesis functional gene *Ej4CL1* promoter and intensifying fruit lignification under low temperatures [91]. In contrast, the loquat transcriptional repressor EjAP2-1 interacts with EjMYB1 and EjMYB2, inhibiting the lignin-related gene Ej4CL1 and reducing lignin synthesis to mitigate fruit lignification during cold storage [92].

### Regulation of stilbenoids and tannins biosynthesis

Resveratrol, a health-promoting stilbene secondary metabolite in grapevine and other plants, accumulates in response to pathogens and confers protective effects in humans. Stilbene synthase (STS), the pivotal enzyme involved in the biosynthesis of resveratrol, has been characterized in a limited number of plant species. Functional characterization revealed that *Vitis quinquangularis* VqERF114 indirectly regulates stilbene biosynthesis. Transient overexpression of *VqERF114* in grape leaves elevated *STS* gene expression and stilbene accumulation. However, Y2H and bimolecular fluorescence complementation assays (BiFC) demonstrated that VqERF114 does not bind *VqSTS* promoters but physically interacts with MYB TF VqMYB35. Crucially, VqMYB35 directly activates *VqSTS15*, *VqSTS28*, *VqSTS42*, and *VqSTS46* by binding their *MBS* promoter. Co-overexpression of *VqERF114* and *VqMYB35* synergistically enhanced *VqSTS* transcription and stilbene production [93]. The ERF family of TFs plays a pivotal role in regulating tannin biosynthesis, particularly during fruit deastringency. In persimmon (*Diospyros kaki*), hypoxia or high-CO₂ treatment triggers ERF-mediated transcriptional activation of key enzymes involved in acetaldehyde metabolism. Specifically, DkERF9 and DkERF10 directly bind to promoters of *DkADH1* (alcohol dehydrogenase) and DkPDC2 (pyruvate decarboxylase), enhancing their expression to promote acetaldehyde accumulation [94]. This process drives the insolubilization of soluble condensed tannins (SCTs), thereby reducing fruit astringency. Mechanistically, DkERF24 interacts synergistically with the WRKY TFs DkWRKY1, forming a heteromeric complex that amplifies DkPDC2 promoter activity through direct binding and protein–protein interactions. Transient overexpression of DkERF24 and DkWRKY1 in persimmon fruit discs significantly upregulates *DkPDC2* transcripts and maintains tannin insolubility. This ERF-WRKY regulatory module exhibits evolutionary conservation, as evidenced by analogous interactions between *Arabidopsis* homologs AtERF1 and AtWRKY53, which similarly transactivate the DkPDC2 promoter [95]. Furthermore, ERFs coordinate with MYB TFs (e.g. DkMYB10) to establish hierarchical regulatory cascades. DkMYB10 activates DkERF9 expression, while DkERF18/19 and DkMYB6 reciprocally regulate DkERF19, forming a feed-forward loop that amplifies hypoxia-responsive signaling. Transient overexpression of these TFs in persimmon fruit tissues corroborates their functional redundancy in promoting SCT insolubilization [93].

### Regulation of shikonin and chalcone biosynthesis

Shikonin is a secondary metabolite accumulated in the roots of the boraginaceae plant *Lithospermum erythrorhizon*, exhibiting antibacterial, anti-inflammatory, and antitumor activities [96]. Studies have found that red, white, and blue light all significantly downregulate the expression of LeERF-1, though the inhibitory effect of red light is relatively weaker compared to other light conditions. Tissue-specific expression analysis further revealed that LeERF-1 is primarily expressed in soil-grown roots cultivated in darkness. These patterns align with the influence of different light signals on the formation of shikonin and its derivatives, suggesting that LeERF-1 may act as a positive regulator in shikonin biosynthesis. Three AP2/ERF TFs were identified as positive regulators of *CitCHIL1* expression. Among these, CitERF32 and CitERF33, activated transcription by directly binding to the CGCCGC motif in the promoter region. In contrast, CitRAV1 formed a transcriptional complex with CitERF33, significantly enhancing both activation efficiency and chalcone accumulation [97].

AP2/ERF TFs play a central role in regulating plant phenolic compound biosynthesis by directly binding to target gene promoters or interacting with other transcription factors, thereby precisely coordinating the phenylpropanoid metabolic network and its downstream branch pathways (Table 2). Phenolic compounds, as a class of important plant secondary metabolites featuring hydroxylated aromatic rings, not only provide structural support and stress resistance to plants but also possess broad medicinal value. These factors extensively participate in regulating the synthesis of phenylpropanoids, flavonoids (anthocyanins), lignin, stilbenoids (resveratrol), tannins, and phenolic acids. Within phenylpropanoid metabolism, they exhibit dual regulatory capabilities: for instance, poplar ERF76 and tobacco NtERF13a enhance stress resistance by activating key enzyme genes (e.g. *HCT*, *F3′H,* and *ANS*) or integrating hormonal signals to promote lignin, chlorogenic acid, and flavonoid synthesis, whereas petunia PhERF6 suppresses floral volatile synthesis by competitively inhibiting the DNA-binding domain of EOBI protein. In anthocyanin biosynthesis, multiple AP2/ERF factors (e.g. apple MdERF38, lily LvERF113, pear PcERF5) promote accumulation by activating promoters of key genes (*MYB10*, *UFGT*) or forming activation complexes with MYB/bHLH (e.g. PuERF27-PuMYB10 synergistically activating *PuGSTF12*); conversely, Arabidopsis AtERF4/AtERF8 and pear PpERF9/PpERF105 repress synthesis by inducing repressive MYBs or competitively binding bHLH proteins. In the lignin pathway, AP2/ERF factors can either activate (e.g. *Isatis indigotica* Ii049 and rice OsERF71 upregulating *CCR* and *CAD* genes) or suppress (e.g. poplar PagERF81 directly inhibiting *PagCCR1* and *PagCAD6*) lignin deposition, with poplar PagERF110 additionally reducing lignin synthesis indirectly by activating repressor PagXND1d. For stilbenoid compounds, grape VqERF114 significantly enhances resveratrol production through interaction with activator VqMYB35 despite lacking direct binding to *STS* promoters. In tannin metabolism, persimmon DkERF9/DkERF10 directly activate acetaldehyde metabolism genes (*ADH1* and *PDC2*) to promote tannin precipitation and astringency removal, while DkERF24 amplifies this effect by forming a complex with DkWRKY1. Furthermore, the light-signal-repressed pattern of *Lithospermum erythrorhizon* LeERF-1 aligns with shikonin biosynthesis, and citrus CitERF32/CitERF33 promote chalcone accumulation by binding to the chalcone synthase *CHIL1* promoter. These regulatory mechanisms involve both direct binding of AP2/ERF to cis-elements (e.g. GCC/DRE) and their interactions with MYB, WRKY, and other factors to form multi-layered regulatory networks, highlighting the family's species- and organ-specific roles in phenolic metabolism and providing critical molecular targets for improving crop stress resistance and quality traits. The related species, natural products, and downstream genes of AP2/ERF TFs regulating phenolic compound biosynthesis are summarized in Table 2.

**Table 2:** The list of reported AP2/ERF TFs involved in the regulation of phenolic compound biosynthesis

**Metabolite Class**	**Related compounds**	**Species**	**AP2/ERF TFs**	**Downstream genes**	**References**
**Flavonosids**	Proanthocyanidins	*Malus domestica*	MdERF1B	MYB9, *MYB11*	[98]
			MdRAV1	MdABI3, MdABI4	[99]
	Anthocyanins	*Pyrus* spp.	Pp4ERF24/96	*MYB114*, *PpUFGT*	[70]
		*Morus alba*	MaERF5	MYBA, F3H	[71]
		*Lilium* spp.	LhERF4	F3H, CHS, CHI	[81]
		*Pyrus ussuriensis*	PuERF27	PuMYB10, PuGSTF12	[12]
		*Malus spectabilis*	MsERF17	MsbHLH3, MsF3’H	[73]
		*Lilium Viviana*	LvERF113	LvMYB1	[74]
		*Pyrus communis*	PcERF5	PcDFR, PcANS, PcUFGT	[75]
		*Solanum melongena*	SmERF118	SmCHS, SmDFR, SmMYB1	[76]
		*Pyrus pyrifolia*	PpERF9	PpRAP2.4, *PpMYB114*	[80]
		*Zanthoxylum bungeanum*	ZbERF3	ZbMYB17	[77]
		*Malus domestica*	MdERF1B	MdMYB9, MdMYB1, MdMYB11	[98]
			MdERF38	MdMYB1	[72]
			MdERF109	MdLNC499	[78]
	Flavonols	*Morella rubra*	MrERF4	MrFLS2	[100]
		*Citrus sinensis*	CsERF003	CHS, CHI	[101]
		*Malus domestica*	MdAP2–34	MdF3’H	[48]
	Chalcone	*Citrus reticulata*	CitERF32/33	CitCHIL1	[97]
	Shikonin	*Lithospermum erythrorhizon*	LeERF-1	-	[102]
**Phenylpropanoids**	Lignin	*Arabidopsis thaliana*	AtERF041	F5H1	[103]
		*Populus alba×P. glandulosa*	PagERF81	PagCCR1	[85]
			PagERF110	PagXND1d	[86]
		*Oryza sativa*	OsERF34	CESA, PAL, CAD	[89]
			OsERF71	PAL, C4H, CCR, CAD, PRX	[90]
		*Citrus sinensis*	CsERF1B	COMT1L, POD, LAC7L	[87]
		*Eriobotrya japonica*	EjAP2–1	EjMYB1/2	[92]
			EjERF39	Ej4CL1	[13]
		*Ipomoea batatas*	IbRAP2.4	CAD, CAR, COMT, C4H	[13]
	Lignan	*Isatis indigotica*	Ii049	PAL, CCL	[88]
	HCAAs	*Arabidopsis thaliana*	ORA59	AtACT	[104]
	Phenolic	*Salvia miltiorrhiza*	SmERF1L1	SmDXR	[34]
**Stilbenoids**	Resveratrol	*Vitis quinquangularis*	VqERF114	VqMYB35, VqSTS15, VqSTS28	[93]
		*Solanum lycopersicum*	SlERF.G3-Like	*SlACO1, SlACS2*	[66]
**Tannins**	-	*Diospyros kaki*	DkERF9/10	DkADH1	[94]
			DkERF24	DkWRKY1	[95]
**Salicylate**	-	*Zea mays*	ZmERF061	ZmMPK6–1	[105]
	-	*Citrus sinensis*	CsERF098	CsSAMT, CsSABP2	[106]

- indicates that the downstream regulatory genes have not been reported.

In the regulation of phenolic compound biosynthesis, the role of AP2/ERF transcription factors is similarly critical and mechanistically diverse. They not only directly regulate key genes in the phenylpropanoid pathway (e.g. *PAL*, *4CL*, *CHS*) but also exhibit a more sophisticated mode of synergistic regulation in the synthesis of flavonoids, including anthocyanins and flavonols, by forming regulatory complexes with other transcription factors such as MYB and bHLH. Compared to terpenoid regulation, this cooperativity based on protein–protein interactions appears more prevalent in phenolic metabolism, highlighting its advantage in modulating complex, branching pathways. Thus far, we have examined two major classes of secondary products derived from carbohydrate metabolism. However, a third crucial component of the plant secondary metabolic landscape remains: the nitrogen-containing alkaloids. These compounds, which utilize amino acids as precursors, possess distinct biosynthetic pathways and chemical properties. In the following section, we will focus on how AP2/ERF transcription factors, particularly members of the ORCA subfamily, have evolved specific regulatory functions to direct the biosynthesis of prominent alkaloids such as nicotine, vinblastine, and camptothecin.

## Regulation of alkaloid biosynthesis by AP2/ERF TFs

**Figure 6:**
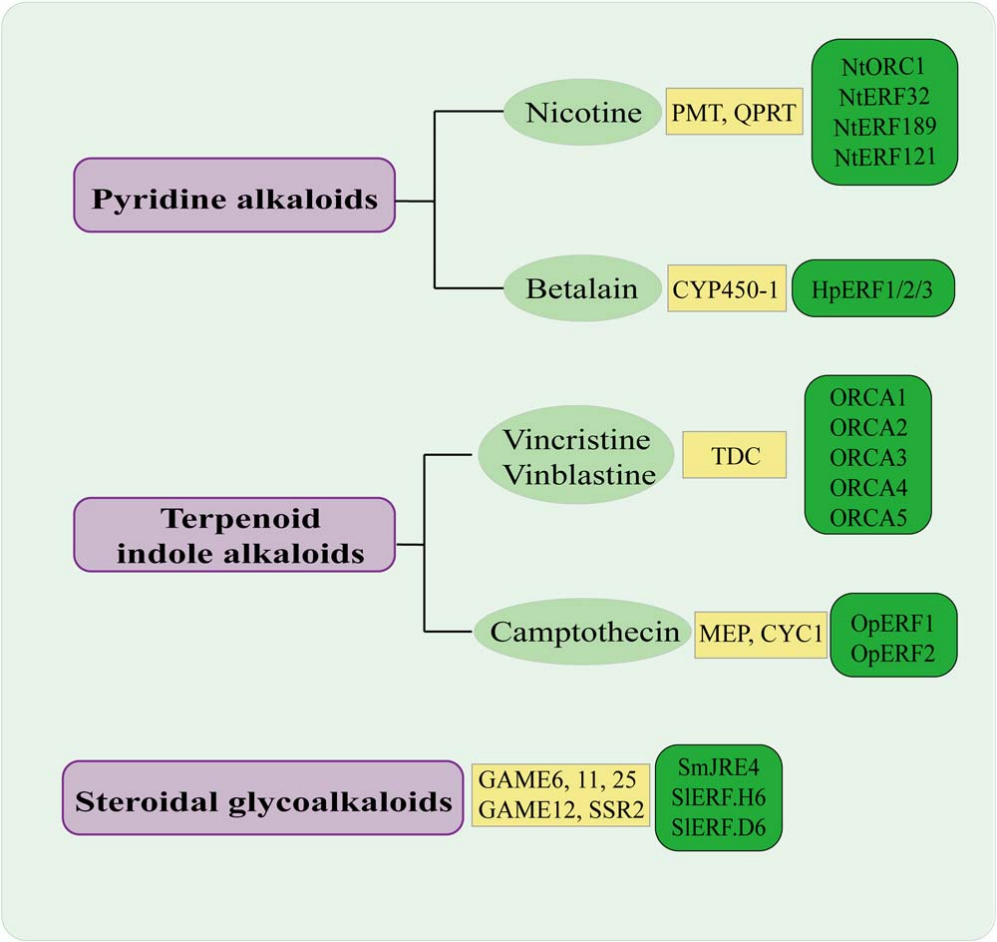
Major classifications of alkaloids and reported AP2/ERF TFs involved in their biosynthesis. This figure summarizes the regulatory roles of AP2/ERF transcription factors in the biosynthesis of several major classes of alkaloids. It highlights three principal categories: Pyridine alkaloids (represented by Nicotine), Terpenoid indole alkaloids (represented by Vincristine/Vinblastine and Camptothecin), and Steroidal glycoalkaloids. The green boxes contain the names of key AP2/ERF TFs that have been experimentally identified as regulators for the corresponding compounds, such as NtORC1 in nicotine synthesis and the ORCA family members in the biosynthesis of alkaloids in *C. roseus*.

Alkaloids are a class of nitrogen-containing organic compounds widely distributed in nature, primarily found in plants (e.g. poppy, cinchona, tobacco) and occasionally derived from animals (e.g. batrachotoxin in poison dart frogs) or microorganisms. Their molecular structures typically feature nitrogen-containing heterocycles (e.g. isoquinoline, indole, pyridine) or amino groups, exhibiting alkaline properties that enable easy salt formation with acids, along with diverse physiological activities. Chemically classified into categories such as isoquinoline alkaloids (e.g. morphine, berberine), indole alkaloids (e.g. vincristine), and quinoline alkaloids (e.g. quinine), they have extensive pharmaceutical applications: morphine and codeine act as analgesics, quinine serves as a classic antimalarial agent, vincristine and paclitaxel are utilized in cancer therapy, while atropine functions as an antispasmodic and pupil-dilating agent. However, certain alkaloids exhibit high toxicity—aconitine induces cardiac arrest, coniine causes respiratory failure, and plants like Datura and Gelsemium contain lethal doses [107]. Biosynthetically, alkaloids originate from amino acids (e.g. tyrosine, tryptophan, lysine) or primary metabolites like mevalonic acid through multi-step enzymatic reactions [108].Isoquinoline alkaloids derive from tyrosine via decarboxylation and cyclization to form dopamine, which condenses with aldehydes to generate benzylisoquinoline intermediates. Indole alkaloids begin with tryptophan, progressing through tryptamine formation and subsequent fusion with terpenoid or secologanin units (as seen in vincristine synthesis). Key steps involve catalysis by oxidases, methyltransferases, and cyclases, followed by post-modifications (e.g. hydroxylation, acetylation, glycosylation), ultimately yielding structurally diverse alkaloids (Fig. 6). This biosynthetic complexity directly determines their distinct physiological activities and functional specificity.

### Regulation of pyridine alkaloid biosynthesis

Pyridine alkaloids are a class of naturally occurring organic compounds characterized by a core aromatic pyridine ring composed of five carbon atoms and one nitrogen atom. They are widely distributed in the plant kingdom and exhibit diverse biological activities [109]. Representative members of these alkaloids include nicotine (primarily derived from tobacco, which stimulates nicotinic acetylcholine receptors to produce stimulant and addictive effects), anabasine (structurally similar to nicotine but containing a piperidine ring, used in addiction mechanism research), ricinine (found in castor beans, causing neurotoxic reactions at high doses), trigonelline (present in coffee beans and fenugreek, acting as a methylated derivative of nicotinic acid with potential antidiabetic properties), and arecoline (the main active component in betel nuts, associated with oral cancer risk and exhibiting cholinergic activity) [110]. Their biosynthesis typically centers on nicotinic acid as a core precursor, generated through the aspartate or kynurenine pathways, and combines with various metabolic fragments (e.g. ornithine-derived pyrrolidine rings) to form complex structures. Ecologically, these compounds often serve as chemical defense agents in plants, protecting against herbivore predation.

Nicotine, also known as tobacco alkaloid, is the primary active component in tobacco. Its biosynthesis begins with the decarboxylation of precursor substances ornithine or arginine by decarboxylase to produce putrescine. Putrescine is then methylated by putrescine N-methyltransferase (PMT) to form N-methylputrescine, which is subsequently oxidized by N-methylputrescine oxidase (MPO) to yield 4-methylaminobutanal. This intermediate combines with aspartate via the pyridine nucleotide cycle pathway to generate nicotinic acid. Through further reactions, nicotinic acid forms a pyridinium intermediate that undergoes natural cyclization to produce pyrrolidine, which ultimately condenses to form nicotine. In tobacco treated with methyl jasmonate, the expression levels of the AP2/ERF TFs NtJAP1 and NtORC1 are upregulated, significantly enhancing the promoter activity of the key downstream enzyme PMT in nicotine biosynthesis. This leads to increased nicotine accumulation, suggesting that NtJAP1 and NtORC1 positively activate nicotine biosynthesis. The expression levels of *NtERF1*, *NtERF32*, and *NtERF121* are quickly heightened in response to MeJA treatment, indicating a rapid activation of these transcription factors. Furthermore, the ectopic overexpression of *NtERF32* has been shown to result in an increase in the expression levels of *NtPMT1a* when examined in vivo. This enhancement goes hand in hand with a rise in the overall alkaloid content, suggesting that NtERF32 may have a significant impact on alkaloid synthesis. Conversely, when *NtERF32* is knocked down using RNAi, there is a noticeable decrease in the expression levels of various genes implicated in the nicotine biosynthetic pathway, including both *NtPMT1a* and quinolinate phosphoribosyltransferase. This reduction not only influence the expression of these critical biosynthesis-related genes but also leads to a decrease in both nicotine and total alkaloid levels, highlighting the vital role of NtERF32 in regulating nicotine production. Overexpression of the AP2/ERF TFs *NtERF189* and *NtERF199* in tobacco results in elevated nicotine levels in leaves, while their knockout mutants show significantly reduced nicotine content [111]. Similarly, the AP2/ERF TFs ORC1, isolated from *Catharanthus roseus*, positively activate structural genes encoding enzymes involved in nicotine biosynthesis. Overexpression of ORC1 in cultured tobacco roots stimulates nicotine production. Notably, nicotine yields are markedly enhanced when the ORC1 promoter contains both the CCC motif and GCC-box. These findings highlight the critical role of AP2/ERF TFs in the nicotine biosynthesis pathway in tobacco.

### Regulation of terpenoid indole alkaloids biosynthesis

*Catharanthus roseus*, a perennial herbaceous plant belonging to the Apocynaceae family, contains over 130 alkaloids, most of which are terpenoid indole alkaloids (TIAs), including vinblastine and vincristine, etc. These compounds were used in the treatment of diseases like lymphoma and leukemia. However, TIAs are present in very low quantities in *C. roseus*. The TIAs metabolic pathway in *C. roseus* is highly complex and tightly regulated. The TIAs biosynthetic pathway was divided into upstream and downstream pathways. In the upstream biosynthetic stage, strictosidine is synthesized via the condensation of tryptamine—produced through the indole pathway via a series of enzymatic reactions—and secologanin—derived from the terpenoid pathway—catalyzed by strictosidine synthase (STR). In the downstream stage, strictosidine serves as the common precursor and undergoes multiple enzymatic reactions to form vindoline and catharanthine, which are ultimately coupled by the newly cloned and characterized class III peroxidase (CrPrx1) to produce vinblastine. In the biosynthesis of vinblastine, several key enzymes involved in both upstream and downstream stages have been extensively studied. These include tryptophan decarboxylase (TDC) and strictosidine synthase (STR) in the upstream stage, as well as desacetoxyvindoline 4-hydroxylase (D4H), deacetylvindoline 4-O-acetyltransferase (DAT), and class III peroxidase (CrPrx1) in the downstream stage. These enzymes play crucial roles in the synthesis of vinblastine and its precursors.

AP2/ERF TFs extensively studied for their regulatory roles in TIAs biosynthesis, the octadecanoid-responsive Catharanthus AP2/ERF (ORCA) family—including ORCA1, ORCA2, and ORCA3. Silencing the *ORCA1* gene upregulates the expression of seven pathway genes related to TIA synthesis and increases TIA accumulation, suggesting that *ORCA1* may act as a negative regulator in TIA biosynthesis. Overexpression of *ORCA2* in hairy roots alters the expression of key genes in both upstream and downstream TIA biosynthetic pathways and significantly affects TIA metabolite accumulation, indicating its crucial role in TIA metabolic regulation. Similarly, overexpression of *ORCA3* in *C. roseus* hairy roots enhances TIA production and upregulates key biosynthetic genes, including *AS*, *TDC*, *DXS*, *CPR*, *G10H*, *STR*, *SGD*, and *D4H*. ORCA4 demonstrates a broad capacity to activate transcription. The introduction of ORCA4 into the hairy roots of *C. roseus* resulted in a remarkable enhancement of TIA production, with an impressive increase exceeding 40-fold in tabersonine levels. Moreover, CrMYC2 is recognized as a crucial regulator within the JA-mediated signaling pathway and exerts direct regulatory effects on ORCA3. In contrast, its influence on ORCA4 and ORCA5 appears to be indirect, likely mediated by an unidentified transcription factor. Additionally, both the ORCA gene cluster and CrMYC2 are subject to further regulatory mechanisms involving a CrMAPK signaling cascade. CrERF5 was identified as a potential regulator. Dual-luciferase assays revealed that CrERF5 activates the promoter of the key gene *TDC* with 2.7-fold higher efficiency compared to the control. Transient overexpression of CrERF5 significantly upregulates key genes in both upstream and downstream TIA biosynthetic pathways and increases TIA metabolite levels, whereas silencing CrERF5 downregulates nine key TIA biosynthetic genes and reduces TIA metabolite content [11]. These findings suggest that CrERF5 may function as a positive regulator of TIA biosynthesis.

Camptothecin is a monoterpene indole alkaloid with therapeutic potential against lung cancer, colorectal cancer, cervical cancer, and ovarian cancer [112]. Analyses of gene expression patterns in *Camptotheca acuminata* tissues, phylogenetic trees, co-expression networks with biosynthetic genes, and promoter sequences of key enzyme genes in the CPT biosynthetic pathway revealed that eight AP2/ERF TFs in *C. acuminata* may regulate CPT synthesis. CaERF1, a member of the DREB subfamily within the AP2/ERF family, has been successfully identified in *C. acuminata*. Transient overexpression and silencing of *CaERF1* in the leaves of *C. acuminata*, have provided compelling evidence that this protein serves a positive regulatory role in the biosynthesis of CPT. This regulation is achieved through the induction of specific genes involved in the iridoid pathway, namely CaCYC1 and CaG8O. Utilizing transient transcriptional activity assays, alongside Y1H, have elucidated the mechanism by which CaERF1 exerts its regulatory effects, CaERF1 is capable of transcriptionally activating the expression of *CaCYC1* and *CaG8O*. This activation occurs through the specific binding of CaERF1 to RAA and CEI elements located within the promoter regions of these two genes [113].

AP2/ERF transcription factors play a central regulatory role in plant alkaloid biosynthesis, primarily through activating downstream gene expression in response to signaling pathways such as JA. In tobacco nicotine biosynthesis, MeJA treatment significantly induces the expression of AP2/ERF members, including NtJAP1, NtORC1, and NtERF32. These factors activate the rate-limiting step of pyrrolidine ring formation by binding to GCC-box or CCC/GAG cis-elements in the promoters of key enzyme genes (*NtPMT1a*). Experimental evidence confirms that overexpression of *NtERF32*/*189*/*199* substantially enhances nicotine accumulation, while RNAi silencing suppresses alkaloid biosynthetic genes (*NtPMT1a*, *NtQPT2*, etc.) and reduces nicotine content. Similarly, in Catharanthus roseus, the ORCA family (ORCA2/3/4)—regulated by the JA signaling cascade (CrMYC2-CrMAPK pathway)—activates upstream and downstream genes (e.g. *TDC*, *STR* and D4H) in the TIA biosynthesis pathway to promote precursors of vinblastine. Among these, *ORCA3* overexpression simultaneously upregulates eight key genes including *AS* and *DXS*, while heterologous expression of *ORCA4* increases tabersonine accumulation by 40-fold. CrERF5 positively activates TIA synthesis by specifically activating the TDC promoter (at 2.7-fold efficiency). In camptothecin biosynthesis, CaERF1 drives camptothecin accumulation by binding to RAA/CEI elements in the promoters of *CaCYC1* and *CaG8O* genes, thereby activating key enzymes in the iridoid pathway. These studies systematically reveal the core mechanism by which the AP2/ERF family integrates environmental signals and targets cis-elements of key alkaloid biosynthetic genes to establish multi-layered transcriptional regulatory networks. The relevant species, natural products, and downstream genes of AP2/ERF TFs that govern the biosynthesis of alkaloids are presented in Table 3.

**Table 3:** The list of reported AP2/ERF TFs involved in the regulation of alkaloids biosynthesis

**Metabolite Class**	**Related compounds**	**Species**	**AP2/ERF TFs**	**Downstream genes**	**References**
**Pyridine alkaloids**	Nicotine	*Nicotiana tabacum*	NtJAP1	PMT	[114]
			NtERF189/199	-	[111]
			ORC1	PMT, QPRT	[115]
			NtERF32/121	PMT1a	[116]
	Betalain	*Hypericum perforatum*	HpERF1/2/3	CYP450–1	[117]
**Trpenoid indolealkaloids**	Vincristine/Vinblastine	*Catharanthus roseus*	CR1/2/3/4/5/6	-	[118]
			CrERF5	TDC	[11]
	Camptothecin	*Ophiorrhiza pumila*	OpERF1/2	MEP	[119]
		*Catharanthus roseus*	CaERF1	CaCYC1, CaG8O	[113]
**Steroidal glycoalkaloids**	Steroidal glycoalkaloids	*Solanum lycopersicum*	SlERF.H6	GAME6, 11, 25	[120]
			SlERF.D6	GAME12	[121]
		*Solanum melongena*	SmJRE4	GAME2, SSR2	[122]

- indicates that the downstream regulatory genes have not been reported.

The analysis of alkaloid biosynthesis regulation further solidifies the central status of AP2/ERF transcription factors. Particularly in the synthesis of terpenoid indole alkaloids (TIAs), AP2/ERF TFs, represented by the ORCA subfamily, act as key responders to jasmonic acid signals, precisely controlling the production of anticancer drug precursors like vinblastine by activating genes such as *TDC* and *STR*. Similarly, in the context of pyridine alkaloids, they direct metabolic flux by regulating *PMT*, the rate-limiting enzyme gene in nicotine synthesis. Integrating the regulatory networks of terpenoids, phenolics, and alkaloids, a common theme emerges: AP2/ERF transcription factors function as critical ‘molecular switchboards’ within the plant, integrating hormonal and environmental signals and converting them into specific chemical outputs. Whether through direct promoter binding or the formation of complex regulatory modules with other transcription factors, they provide plants with unparalleled regulatory flexibility for environmental adaptation and chemical defense. These findings not only deepen our understanding of plant metabolic regulation but also illuminate future directions for metabolic engineering applications.

## Conclusions and prospects

This review provides a comprehensive summary of the crucial regulatory roles played by AP2/ERF transcription factors (TFs) in the biosynthesis of major classes of plant natural products, such as terpenoids, phenolic compounds, and alkaloids. A growing body of evidence suggests that AP2/ERF TFs serve as central hubs, integrating various developmental cues and environmental signals (e.g. phytohormones like JA and ET, abiotic/biotic stresses) to precisely coordinate secondary metabolic pathways. These TFs employ a range of molecular mechanisms, including direct promoter binding (e.g. to GCC-box, DRE/CRT elements), indirect regulation through signaling cascades or intermediate TFs, co-regulation through protein–protein interactions (e.g. with MYB, bHLH, WRKY factors), and self-regulation via feedback loops or post-translational modifications. This intricate control allows plants to dynamically allocate resources, optimize defense responses, and adapt to changing environments, all while producing a vast array of structurally complex metabolites with significant ecological, pharmaceutical, and industrial value.

Since the first cloning of AP2/ERF TF in *A. thaliana*, significant progress has been made in studying these regulators, particularly regarding their roles in plant growth, development, and environmental stress responses. However, several challenges remain unresolved. Firstly, current functional studies of AP2/ERF TFs predominantly focus on model plants such as Arabidopsis, tomato, and rice, with limited research on their regulatory roles in medicinal plants' bioactive secondary metabolites. Secondly, existing investigations mainly concentrate on individual TFs' isolated functions, while the interaction mechanisms between different signaling pathways and synergistic effects with other TFs require further exploration. Notably, some tandem-arranged AP2/ERF TFs exhibit functional redundancy, making it difficult to elucidate their mechanisms using traditional molecular biology techniques. Additionally, although numerous studies have characterized specific TFs, practical applications for developing stable high-yield medicinal plant strains to address insufficient sources of bioactive components—particularly rare natural products—remain scarce. These limitations stem from two main aspects: the inherent complexity of plant secondary metabolism influenced by various internal and external factors, and the incomplete understanding of secondary metabolic pathways in most medicinal plants.

The interplay between AP2/ERF transcription factors and epigenetic regulation represents an emerging frontier, adding a new layer of complexity to the regulatory networks of secondary metabolism. Future research should aim to elucidate how epigenetic modifications, such as DNA methylation and histone modifications (e.g. H3K27ac, H3K4me3), influence the expression of AP2/ERF genes themselves, potentially enabling them to respond rapidly to developmental or environmental cues. Conversely, investigating whether AP2/ERF TFs can act as scaffolds to recruit chromatin-modifying complexes—such as histone acetyltransferases (HATs) or deacetylases (HDACs)—to the promoter regions of biosynthetic genes would provide deeper mechanistic insights. This reciprocal relationship, where epigenetics regulates the regulators and the regulators in turn modify the epigenetic landscape, may play a critical role in establishing stable yet plastic metabolic phenotypes and could serve as an important target for future crop improvement strategies.

TFs serve as crucial tools in regulating plant secondary metabolism through metabolic engineering to produce bioactive compounds. Compared to individual catalytic enzymes, TFs exert comprehensive and efficient control over secondary metabolic pathways while simultaneously influencing the growth and development of organs involved in secondary metabolite synthesis and promoting their accumulation. Therefore, research on TFs has consistently been a key focus for manipulating complex metabolic networks and obtaining abundant bioactive natural products. The AP2/ERF transcription factors represent powerful tools for metabolic engineering and synthetic biology, offering significant advantages for enhancing the production of high-value natural products. Unlike the modification of single biosynthetic enzymes, which can create metabolic bottlenecks or imbalances, targeting a master regulator like an AP2/ERF TF allows for the coordinated and comprehensive modulation of an entire pathway. This makes them ideal candidates for engineering strategies aimed at improving crop quality and increasing the yield of valuable phytochemicals. In synthetic biology, AP2/ERF TFs can be repurposed as tunable ‘molecular switches’ or biosensors within engineered genetic circuits. By placing them under the control of inducible promoters or engineering their ligand-binding domains, their activity can be precisely controlled in response to specific chemical inducers, allowing for dynamic regulation of metabolic flux. Furthermore, protein engineering approaches can be used to modify the DNA-binding or activation domains of AP2/ERF TFs to create novel synthetic regulators with altered target specificity or enhanced potency, thereby enabling the precise redirection of metabolic pathways toward desired products.

A critical future direction is to systematically unravel the evolutionary mechanisms driving the functional divergence of the AP2/ERF family across plant lineages, which in turn requires constructing more comprehensive secondary metabolic regulatory networks by revealing upstream regulators, regulatory mechanisms, and inter-TF interactions. This necessitates moving beyond single-species studies to comparative analyses that explore how gene family expansions and contractions—such as the ERF subfamily’s proliferation in tobacco or the loss of Soloist members in rice—have shaped specialized metabolic networks. To this end, future research could test specific, forward-looking hypotheses: for instance, that expansions of certain ERF subclades are directly linked to the emergence of lineage-specific metabolic pathways, or that the functional divergence between orthologs (e.g. the broad stress-response role of *Arabidopsis* AP2/ERF TFs versus the highly targeted regulation by *S. miltiorrhiza* AP2/ERF TFs) is driven by promoter evolution and changes in chromatin accessibility of their target genes.

Testing these hypotheses will demand an integrative approach that leverages recent advancements in high-throughput sequencing, synthetic biology, and computational biology for TF discovery, functional annotation, and pathway design. Specifically, combining comparative genomics and phylogenomics with cutting-edge functional assays is essential to dissect and engineer these regulatory networks with greater precision. For example, the application of CRISPR/Cas9-based tools, particularly CRISPR activation (CRISPRa) and CRISPR interference (CRISPRi), offers a powerful method for modulating endogenous AP2/ERF genes in their native chromatin context, thus avoiding the artifacts of traditional methods and enabling a more accurate assessment of their functions. Furthermore, the integration of multi-omics approaches is crucial for building comprehensive regulatory models; single-cell and spatial transcriptomics can resolve cell-type-specific expression patterns, and combining these maps with chromatin accessibility data from techniques like ATAC-seq will allow for the genome-wide identification of direct AP2/ERF targets. Ultimately, these advanced methods, alongside cross-species complementation experiments, will be crucial for validating the functional consequences of evolutionary changes in both the transcription factors and their target cis-elements, providing a systems-level understanding of the complex regulatory architecture they govern.

## Supplementary Material

Web_Material_uhaf280
