# Supplementary material for: AP2/ERF transcription factors regulate the biosynthesis of terpenoids, phenolics, and alkaloids in plants
Source: Hortic Res. 2025 Oct 20;13(1):uhaf280. doi: 10.1093/hr/uhaf280 (PMC12871079; doi:10.1093/hr/uhaf280)
Supplement: Web_Material_uhaf280 [file web_material_uhaf280.docx]

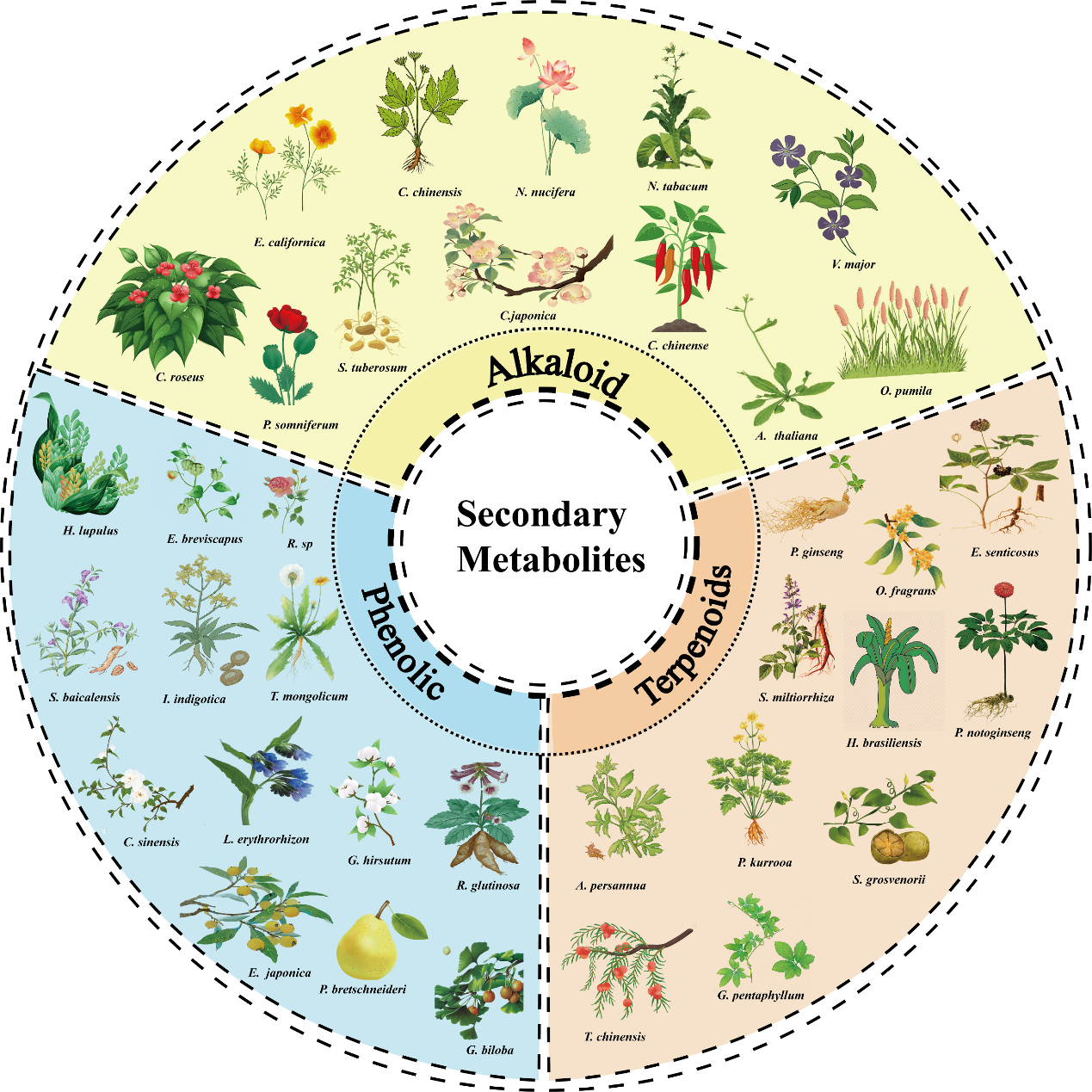


**Fig. S1 Plant species in which AP2/ERF TFs have been reported to be involved in the regulation of secondary metabolism.** This figure displays a collection of plant species where AP2/ERF transcription factors have been experimentally confirmed to participate in the regulation of secondary metabolite biosynthesis. The plants are categorized into three major classes based on the primary type of secondary metabolite studied: Terpenoids, Phenolics, and Alkaloids. The classification of each species is based on functional validation evidence from published literature.
